# Overdose of dolutegravir in combination with tenofovir disaproxil fumarate/emtricitabine in suicide attempt in a 21-year old patient

**DOI:** 10.1186/s12981-015-0054-y

**Published:** 2015-05-21

**Authors:** Marcel Lee, Florian Eyer, Norbert Felgenhauer, Hartwig HF Klinker, Christoph D Spinner

**Affiliations:** Department of Clinical Toxicology, University Hospital Klinikum rechts der Isar, Technische Universität München, Ismaningerstr. 22, Munich, 81675 Germany; Department of Medicine II, Division of Infectious Diseases, University Of Wuerzburg Medical Center, Oberduerrbacherstr. 6, Wuerzburg, 97080 Germany; Department of Medicine II and Interdisciplinary HIV Center (IZAR), University Hospital Klinikum Rechts Der Isar, Technische Universität München, Ismaningerstr. 22, Munich, 81675 Germany

**Keywords:** HIV, AIDS, Suicide attempt, Dolutegravir, Tenofovir disaproxil fumarate, Emtricitabine, Overdose

## Abstract

A 21 year old MSM patient with newly diagnosed HIV infection was hospitalized in our department after ingestion of an overdose of his antiretroviral therapy (ART) comprising dolutegravir (DTG - Tivicay®) and tenofovir disaproxil fumarate/emtricitabine (Truvada®) in suicidal intention. On admission, the patient did not show any clinical signs of intoxication and laboratory findings were unremarkable. After 6 hours of intensive care monitoring, the patient was referred to a psychiatric clinic. 5 days after the day of intoxication, serum creatinine levels increased to high normal values (1.2 mg/dl). However, levels never exceeded the upper threshold. 8 and 12 weeks later, serum creatinine normalized to levels measured prior to the intoxication. No other adverse events occurred, and the patient does not suffer from permanent impairments.

## Background

Antiretroviral therapy is employed in HIV patients to achieve durable suppression of HIV replication, thereby controlling HIV RNA, to restore immune function and to reduce morbidity and mortality, to improve quality of life, as well as to limit the likelihood of viral resistance and reduce the risk of viral transmission [1-3]. Dolutegravir (DTG) is a novel second-generation integrase inhibitor and has recently been introduced for use as antiretroviral therapy of HIV infection [4]. Recommended dose for treatment of therapy-naïve patients is 50 mg per day. DTG is known to be an inhibitor of the renal transport protein organic cation transporter 2 (OCT2), resulting in a reversible elevation of serum creatinine [5]. Furthermore, inhibition of the OCT2 can result in relevant drug interactions, e.g. dofetilide, metformin, cimetidine, triamterene, and procainamide [6].

## Case presentation

We report a 21-year old MSM patient who was admitted to our department on June 2014 following an overdose of antiretroviral medication (ART) in suicidal intention. HIV-infection was diagnosed 3 weeks prior to his admission to our department.

CDC-stage of the HIV infection at primary diagnosis was A1, WHO stage I with a documented CD4-nadir of 590/μL (norm: 490 – 1760/μl), CD4/CD8 ratio of 0.7 (norm 1.0 – 5.8) and an HIV load of 42.400 cps/ml. HIV diagnostic was performed because of a syphilis stage II infection with a generalized rash, a couple of weeks before. The syphilis infection was treated with doxycycline 200 mg per day for 14 weeks and due to incomplete decrease of VDRL titers followed by intramuscular benzathine penicillin 2.4 million units on day 0, 7, 14. With the patient’s consent an ART with DTG (Tivicay®) and tenofovir disaproxil fumarate/emtricitabine (TDF/FTC - Truvada®) was initiated immediately.

His medical history includes an allergic bronchial asthma, which is treated with ipratropium bromide and fenoterol hydrobromide. The patient reported on request occasional cocaine consumption (recreational drug abuse).

On day of admission, the patient reports to have taken 15–20 pills of TDF/FTC and the same amount of DTG, amounting to a maximum of 4900 mg TDF/ 4000 mg FTC and 1000 mg DTG in suicidal intention. He admitted having combined the ART overdose with an unknown amount of vodka and cocaine. Exact time of ingestion was unclear but most likely within the past 4 hours prior to admission. Physical examination revealed minor airway obstruction; the remaining examination was without pathological findings. The patient did not specify any complaints. He was hospitalized and monitored for 6 hours on our intensive care unit. Within that period of time, no adverse events occurred, no specific therapy was necessary.

**Figure 1:**
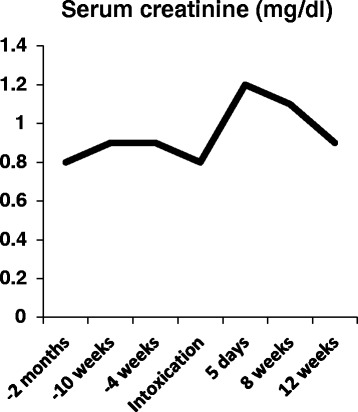
**Course of serum-creatinine concentration starting two months prior to intoxication, day of intoxication and follow up period.** The transient elevation of serum creatinine five days after intoxication and the following normalization are demonstrated.

Upon admission a serum sample was routinely taken. Blood analysis revealed a minimal TSH-elevation while normal free thyroid hormons were documented (TSH 4.30 μIU/ml, fT3 4.0 pg/ml, fT4 1.4 ng/ml) (Norm: TSH 0.27 – 4.2 μIU/ml, fT3: 2.00 – 4.30 pg/ml, fT4: 0.90 – 1.90 ng/ml), which was discussed as latent hypothyroidism formally. The remaining parameters showed no pathological findings. Electrocardiographic analysis showed no pathological findings, especially QTc time was normal. DTG concentration in serum sample was 10,013 ng/ml (norm: > 500 ng/ml). To date, the analyzing lab did not establish upper limits, however the IC90 for DTG is 64 ng/ml [7], and the mean plasma concentration from 102 samples acquired from 65 patients analyzed in our lab was 2,226 ng/ml (+/−1,693 ng/ml). These data justify the conclusion that there was a relevant overdosing in our patient, measured approximately 4 hours after ingestion. Toxicological analysis revealed an ethanol concentration of 0.48 g/l. Intracellular TDF- and FTC-levels could not be monitored, because no cell fraction sample was stored at time of admission. Creatinine concentration on admission was 0.8 mg/dl (70.72 μmol/l) (range: 0.5 – 1.3 mg/dl; 44.2 – 114.92 μmol/l). Five days after ingestion, creatinine concentration increased 50 % to 1.2 mg/dl (106.08 μmol/L), however, the parameter never exceeded the pathologic threshold. Subsequent analysis of serum creatinine eight weeks later revealed slight reduction to 1.1 mg/dl (97.24 μmol/l), analysis 12 weeks later revealed further reduction of serum levels to 0.9 mg/dl (79.56 μmol/l), representing his baseline serum creatinine. (Figure 1) No evidence for any proteinuria or Fanconi syndrome was observed during observation’s period.

The patient was discharged after uneventful monitoring from the hospital without any symptoms and TDF/FTC + DTG ART was continued without any changes. He was followed up in the HIV outpatient center and psychiatric therapy was offered.

## Conclusion

Our case of a 21-year old HIV-patient ingesting an overdose of DTG and TDF/FTC in suicidal intention suggests a broad safety range of the above mentioned HIV drugs in the overdose setting of an otherwise healthy patient. As has been reported, DTG is a potent inhibitor of the renal transporter OCT2 with creatinine accumulation resulting from that effect [5,8]. In combination with the potential nephrotoxic TDF, this could lead to impaired renal function in case of overdose with one or both drugs. To the best of our knowledge, additional effects of TDF overdose or TDF and DTG overdose with possible implications on renal function of both drugs have not been reported so far [9]. However, previous studies demonstrated that the elevation of creatinine following DTG ingestion is not a consequence of reduction in renal blood flow or renal creatinine clearance (i.e. no nephrotoxic effect), but is rather directly attributable to the inhibition of OCT2. This effect was found to be reversible [10].

The clinical course of our patient, showing an increase in creatinine baseline levels of 50% within the first five days after ingestion, but recovering to previous levels after the acute event, support the current safety data of DTG leading to an intermittent increase in serum creatinine. No permanent impairment in renal function remained in our case.

In conclusion, DTG over dosage does not seem to lead to sustained nephrotoxic effects in our case, which supports the previous study findings of the favorable safety profile of the drug. Further data from routine clinical use and safety studies are needed to confirm these findings in case of DTG overdose.

## Consent

Written informed consent was obtained from the patient for the publication of this report and any accompanying images.

## Abbreviations

ART: Antiretroviral therapy
DTG: Dolutegravir
FTC: Emtricitabine
OCT2: Organic cation transporter 2
TDF: Tenofovir disaproxil fumarate
